# Ileocolic Intussusception Secondary to Appendicular Mass in an Adult Patient: A Case Report and Radiological Approach

**DOI:** 10.7759/cureus.46739

**Published:** 2023-10-09

**Authors:** Ahmad E Alhejji, Eman Al Khalifah, Ali Alhejji, Hani M Al Salam, Mohammed A Alobaid

**Affiliations:** 1 Radiology, Qatif Central Hospital, Qatif, SAU; 2 Ophthalmology, King Abdulaziz University Faculty of Medicine, Jeddah, SAU; 3 Radiology, Omran General Hospital, Al-Omran, SAU

**Keywords:** radiological approach, complicated appendicitis, appendicular mass, intussusception in adults, ileocolic intussusception

## Abstract

Intestinal intussusception is a condition determined as the telescoping of one portion of the bowel loops into the approximate distal portion. Intestinal intussusception is a fairly rare condition in the adult population and almost always secondary to the underlying pathology; the most common leading causes of intussusception in adults are neoplasms, accounting for up to two-thirds of the causes. However, appendicular pathology is an extremely rare leading cause. The clinical picture of an adult patient with bowel intussusception (BI) is uncertain and nonspecific; it varies from vague abdominal pain to clinical presentation of complications, such as signs of bowel obstruction or bowel perforation. Therefore, early access to imaging is the cornerstone for the early detection and establishment of the provisional diagnosis of BI. Herein, we report a case of ileocolic intussusception in an adult patient secondary to appendicular phlegmon; moreover, we propose a radiological approach to reach the diagnosis of intestinal intussusception in the adult age group.

## Introduction

Intestinal intussusception is a condition determined as the telescoping of one portion of the bowel loops into the approximate distal portion [1]. Although it is a common condition in infant and pediatric age groups, it is a fairly rare condition in the adult population, with incidence approximated at two to three cases per 1,000,000 population per year, 5% of all intussusception cases, and 1-5% of obstruction bowel cases [2,3]. 

Intestinal intussusception in adults is predominantly secondary to the underlying pathology; the most common leading causes of intussusception in adults are neoplasms, accounting for up to two-thirds of the causes. However, appendicular pathology is an extremely rare leading cause [4].

Intestinal intussusception can give rise to serious complications, including bowel ischemia, necrosis, and viscous perforation. Therefore, raising high suspicion and early detection is vital to prevent complications and reduce mortality [5,6]. In the present case, we document an incidence of ileocolic intussusception secondary to appendicular phlegmon that was surgically managed with ileocecal resection.

## Case presentation

 A 46-year-old male with a known case of sickle cell disease presented to the emergency room complaining of right lower abdominal pain that started one month ago with on-and-off phenomena that became more severe over the last week. The pain is colicky in nature with no radiation, associated with nausea, vomiting, anorexia, diarrhea, weight loss of 4 kg over the past two weeks, and fever. On physical examinations, the blood pressure was 109/52 mmHg, pulse rate 106 beats/minute, respiration rate 22 breaths/minute, and body temperature 38°C. Abdominal examinations demonstrated no abdominal distention and no skin changes. The abdomen is soft and lax, with tenderness in the right iliac fossa. His laboratory results showed white blood cells of 26.03 x 10^9^/L, hemoglobin 11.1 g/dl, platelets 376 x 10^9^/L, and creatinine 61 µmol/L. 

**Figure 1:**
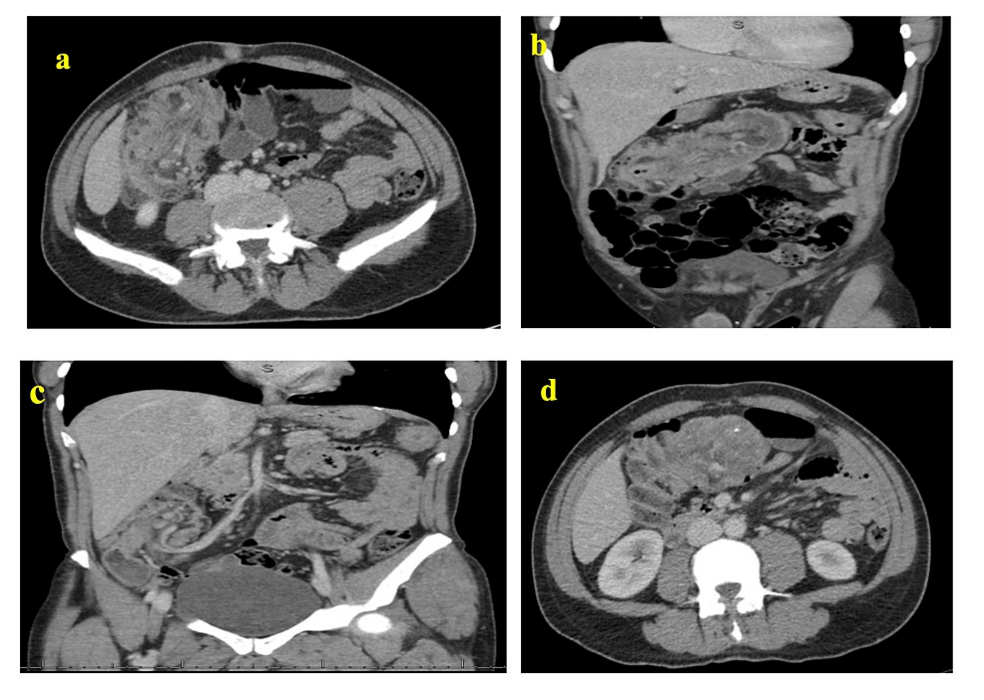
a, b: Axial and coronal CT images with contrast demonstrating a large mass at the right colonic flexure show the cecum and parts of the ileocecal valve, and the adjacent mesenteric fat represents the (intussusceptum) telescoped into the lumen of the transverse colon (intussuscipiens). c: Coronal CT image showing vascular structures twisted toward the mass, including the superior mesenteric vein (SMV) and mesenteric vessels. d: Axial CT image calcification projected inside the mass with a non-visualization of the normal appendix. Projected inside the mass with a non-visualization of the normal appendix.

Computed tomography (CT) scan with IV contrast enhancement of the abdomen and pelvis was done, as shown in Figures 1A, 1B, 1C, 1D. Ileocolic intussusception is noted with telescoping of the cecum/ileocecal valve, including the inflamed appendicular mass with an adjacent mesenteric vessel and fat within the ascending colon. The lead point is the appendicular mass. 

**Figure 2:**
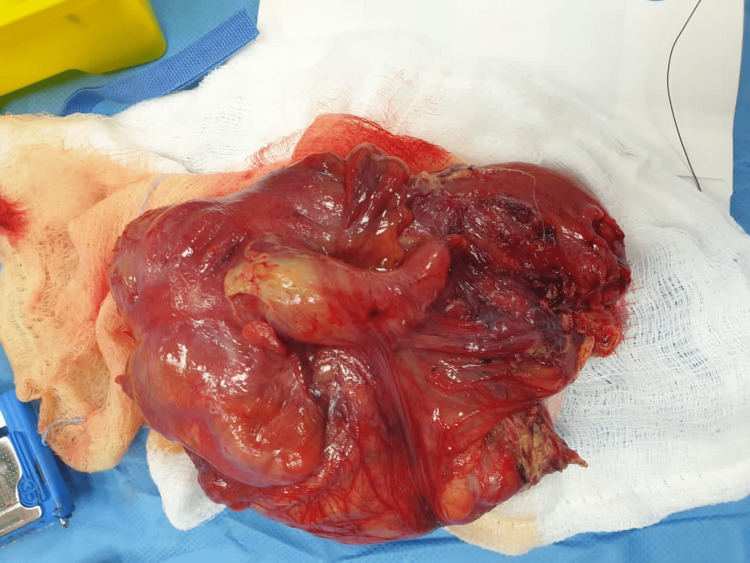
Gross appearance of the resected ileocecal segment with a dilated, inflamed appendix with wall defect, representing the perforated appendix.

The patient was admitted as a case of ileocecal intussusception and shifted to the operation room. The diagnosis of ileocolic intussusception with an inflamed perforated appendix and surrounding inflammatory changes as the lead point was confirmed intraoperatively, and ileocecal resection was done, as shown in Figure 2.

The histopathology showed the appendix with mucosal ulceration, mural edema, mural-dense acute and chronic inflammation, focal fibrosis, focal calcification, bacterial colonies, and severe serositis, consistent with acute appendicitis with peri-appendicitis. Moreover, the laboratory finding show cecum with mural edema and five lymph nodes with reactive follicular lymphoid hyperplasia and sinus histiocytosis. The patient was discharged in good condition after the operation.

## Discussion

Bowel intussusception (BI) is an uncommon condition in the adult population, which can be categorized depending on the presence of a leading point into a leading-point intussusception, which is secondary to the underlying pathology and non-leading-point intussusception [6,7]. The most prevalent leading causes of intussusception in adults are neoplasms, accounting for up to two-thirds of the causes. Small BI in adults is commonly due to benign tumors, while large BI is usually due to malignant tumors [6].

Appendicitis is an extremely rare cause of BI in adults with an incidence rate of approximately 0.01% [8]. A previous case report from the College of Medicine of Taibah University, Saudi Arabia, was published in 2012 by Guraya et al., which talks about a 17-year-old female patient who came to the hospital with a history of right iliac fossa pain and was diagnosed as a case of ileocolic intussusception secondary to uncomplicated acute appendicitis [9]. Another case report from West Virginia University School of Medicine, USA, published in 2019 by Green et al., describes the case of a 42-year-old female patient who presented to the hospital complaining of left lower abdominal pain and left-side ileocolic intussusception secondary to a large mucinous tumor of left-side appendix [10]. The unique thing about the presenting case is that the ileocolic intussusception is secondary to complicated appendicitis by appendicular mass.

BI in adults is associated with a high mortality rate [5]. The clinical picture of intestinal intussusception in adults is uncertain and nonspecific; it varies from vague abdominal pain to the clinical presentation of complications, such as signs of bowel obstruction or bowel perforation. Therefore, early access to imaging is the cornerstone for the early detection and establishment of the provisional diagnosis of BI [3].

Abdominal CT scan with IV contrast enhancement is the gold standard for diagnosing BI. It has a cost-benefit value and is easy to access, with a documented diagnostic accuracy ranging from 58% to 100% [3].

The questions that the radiologist should answer regarding BI in adults are as follows: First, is it intussusception or not? The appearance of BI is described as a bowel-within-bowel pattern, which shows duplicated intestinal layers that appear as concentric rings (the CT counterpart of the sonographic target sign) when viewed perpendicular to the lumen and as a soft tissue sausage when viewed longitudinally. Transient intussusception, primary intestinal tumors, metastatic tumors, intestinal lymphoma, intestinal lipoma (without intussusception), Meckel diverticulum, gallstone ileus (cholesterol stones can have a layered appearance with fat density), and endometriomas are mimickers that may have a similar appearance of BI [3,7].

Second, where is the location? What are the segments of the bowel that are involved? The location could be enteroenteric, ileocolic, ileocecal, or colo-colic [7].

Third, what is the lead point? Although the CT findings that help distinguish between lead-point and non-lead-point intussusception have not been thoroughly studied and there is a significant overlap of the CT findings, a lead mass is seen at CT as a separate and distinct entity as opposed to edematous bowel, so it can be considered a reliable indicator of a lead-point intussusception. Making the distinction between lead-point and non-lead point intussusception is crucial when deciding on the best course of action and can potentially decrease the number of unnecessary surgical operations [4,7].

Finally, and not the least important, is there any complication or not? The complications are the late sequelae of BI; the serious complications are intestinal obstruction, bowel ischemia, and bowel perforation, which may require sophisticated surgeries and are associated with higher mortality rates and post-surgical complications [2,3,5,6].

## Conclusions

This is a case of ileocolic intussusception in an adult patient secondary to appendicular phlegmon. Intestinal intussusception is a rare condition in the adult age group and is almost always secondary to underlying pathology. The diagnosis of intestinal intussusception in adults is challenging for both clinical and radiological aspects; therefore, raising high suspicion, early access to the imaging, and careful assessment of the radiological findings are crucial for early diagnosis and for choosing the proper management plan before complications take place to reduce the mortality and to improve the quality of life.
